# Advancing Pharmacist Collaborative Care within Academic Health Systems

**DOI:** 10.3390/pharmacy7040142

**Published:** 2019-10-11

**Authors:** Linda Awdishu, Renu F. Singh, Ila Saunders, Felix K. Yam, Jan D. Hirsch, Sarah Lorentz, Rabia S. Atayee, Joseph D. Ma, Shirley M. Tsunoda, Jennifer Namba, Christina L. Mnatzaganian, Nathan A. Painter, Jonathan H. Watanabe, Kelly C. Lee, Charles E. Daniels, Candis M. Morello

**Affiliations:** 1 San Diego Skaggs School of Pharmacy and Pharmaceutical Sciences, University of California, La Jolla, CA 92093, USA; rfsingh@ucsd.edu (R.F.S.); isaunders@ucsd.edu (I.S.); fyam@ucsd.edu (F.K.Y.); jdhirsch@uci.edu (J.D.H.); slorentz@ucsd.edu (S.L.); ratayee@ucsd.edu (R.S.A.); jdma@ucsd.edu (J.D.M.); smtsunoda@ucsd.edu (S.M.T.); jnamba@ucsd.edu (J.N.); cmnatzaganian@ucsd.edu (C.L.M.); npainter@ucsd.edu (N.A.P.); Jhwatanabe@ucsd.edu (J.H.W.); kellylee@ucsd.edu (K.C.L.); cdaniels@ucsd.edu (C.E.D.); candismorello@ucsd.edu (C.M.M.); 2 San Diego Health System, University of California, La Jolla, CA 92093, USA; 3 Veterans Affairs San Diego Healthcare System, La Jolla, CA 92093, USA; 4 Irvine School of Pharmacy and Pharmaceutical Sciences, University of California, Irvine, CA 92697, USA

**Keywords:** collaborative practice, clinical pharmacy, advanced practice pharmacist provider

## Abstract

**Introduction**: The scope of pharmacy practice has evolved over the last few decades to focus on the optimization of medication therapy. Despite this positive impact, the lack of reimbursement remains a significant barrier to the implementation of innovative pharmacist practice models. **Summary**: We describe the successful development, implementation and outcomes of three types of pharmacist collaborative care models: (1) a pharmacist with physician oversight, (2) pharmacist–interprofessional teams and (3) physician–pharmacist teams. The outcome measurement of these pharmacist care models varied from the design phase to patient volume measurement and to comprehensive quality dashboards. All of these practice models have been successfully funded by affiliated health systems or grants. **Conclusions**: The expansion of pharmacist services delivered by clinical faculty has several benefits to affiliated health systems: (1) significant improvements in patient care quality, (2) access to experts in specialty areas, and (3) the dissemination of outcomes with national and international recognition, increasing the visibility of the health system.

## 1. Introduction

Collaborative care between pharmacists and physicians has been recognized to improve pharmacotherapeutic outcomes and provide increased value and efficiency to the health care system [1]. Models for collaborative drug therapy management have been designed with a focus on the most studied chronic diseases including hypertension (HTN) and type 2 diabetes (T2D). Numerous studies have demonstrated the positive impact that physician–pharmacist collaborative management can have on blood pressure and glycosylated hemoglobin (A1C) among patients with HTN and DM [2,3,4]. Additionally, this positive impact has been demonstrated for other chronic diseases [5,6]. More recently, Matzke and colleagues demonstrated that pharmacists integrated in a patient-centered medical home reduce hospitalizations by 23.4%, resulting in estimated cost savings of $2619 per patient [7]. Despite the overwhelmingly positive impact of pharmacist services on patient outcomes, the integration of pharmacists in ambulatory care programs is not widespread. A survey conducted by Hattingh and colleagues identified three major themes as factors that impacted the provision of pharmacist services: (1) pharmacist characteristics, (2) local needs, structures and support, and (3) an enabling practice framework [8,9]. In this paper, we describe the program development, successful implementation and outcomes of pharmacist collaborative care models provided by the University of California, San Diego Skaggs School of Pharmacy and Pharmaceutical Sciences (SSPPS) faculty in two affiliated academic health systems.

### Setting

For decades, the scope of pharmacy practice in the United States (US) has expanded to optimize medication use to achieve patient health-related goals. More recently, the passage of California Senate Bill 493 allows all pharmacists to administer drugs and biologics when ordered by a prescriber, furnish oral contraceptives, travel medications and nicotine replacement products for smoking cessation, as well as administer vaccinations. This bill established an Advanced Practice Pharmacist (APP) recognition and authorizes APPs to perform patient assessments, order and interpret drug therapy–related tests, refer patients to other health care providers, initiate, adjust, and discontinue drug therapy upon referral from a patient’s treating prescriber and in accordance with established protocols, and participate in the evaluation and management of diseases and health conditions in collaboration with other health care providers. The APP recognition requires a separate APh licensure application, review and approval from the California Board of Pharmacy.

At UC San Diego Health (UCSDH) and the Veteran’s Affairs San Diego Healthcare System (VASDHS), comprehensive collaborations were developed in which pharmacists provide patient care under collaborative practice agreements (CPA) in the inpatient and ambulatory care settings. The positive impact of pharmacist services has been established using various models of care for chronic disease management [10,11,12]. However, recognition of pharmacists as providers has been challenged, and reimbursement for services has been limited. The lack of reimbursement for services creates a barrier to the implementation of new pharmacist care models in health systems [13].

## 2. Health System Overview

UCSDH is a comprehensive health system with an average daily census of approximately 600 patients and over 580,000 ambulatory care visits per year. Approximately 12 faculty from SSPPS provide collaborative care within the health system. Since 2010, the UCSDH pharmacy department developed a program expanding the role of the pharmacist in inpatient and ambulatory care and affiliated clinic settings. VASDHS provides care to more than 249,594 Veterans in the San Diego and Imperial Valley counties. In addition to the main facility in San Diego, VASDHS has six community-based outpatient clinics. Two faculty from SSPPS provide patient care within VASDHS. Faculty partner with staff pharmacists within each affiliated health system to develop new services. CPAs were developed and implemented for practice areas including chronic kidney disease (CKD), diabetes, family medicine, heart failure (HF), oncology, pain and palliative care, psychiatry, solid organ transplantation (SOT), and transitions of care (TOC). Collaborative care is delivered using three main care models: (1) pharmacists with physician oversight, (2) pharmacist–interprofessional teams, and (3) physician–pharmacist teams.

### 2.1. Needs Assessment and Proposal Development

Each program arose from a clinical need identified by the health system or through a needs assessment conducted by individual faculty with subspecialty expertise. Some needs were based on regulatory requirements (e.g., solid organ transplant), gaps identified by a physician champion, or grant funding available for novel clinical services. Initial steps include proposal development, identified gaps in care, proposed outcome measures, and a business case for return on investment. All pharmacist practices were successfully funded by affiliated health systems or grants.

### 2.2. Implementation

A multi-professional, collaborative approach involving pharmacists, physicians, nurses and administration personnel was required to establish and streamline the patient care process for the target population. Clinical program development included creating a scope of practice and credentialing, clinical guidelines or protocols, clinic schedules and space, referral criteria and documentation templates in the electronic health record (EHR). In each affiliated practice setting, the pharmacist–physician team determined the pharmacist scope of practice. Within the scope of practice, prescribing authorities were defined for specific diseases or conditions. These authorities include the initiation, adjustment and discontinuation of medications according to program-specific clinical guidelines or health system policies, where appropriate. Pharmacists have the authority to order medication-related tests (i.e., laboratory, pharmacogenomic and radiographic tests) to monitor therapy outcomes and make referrals to other providers. The CPA is not limited to specific medications (with the exception of controlled substances, where this is required by the Drug Enforcement Agency), providing prescribing flexibility. All pharmacists who wished to establish a CPA with a physician must have been licensed in California and demonstrate experience and competence treating patients within their specialty in order to be credentialed through their respective health system.

### 2.3. Outcome Measurement

EHRs enable the measurement of patient care clinical and process outcomes. Each program is responsible for defining, measuring and reporting care outcomes. Collaborative practice outcome measurements are in different phases, with some at the design phase and others using comprehensive dashboards. Dashboards are useful tools for tracking outcome measures and identifying areas for performance improvement. However, this approach is largely retrospective and dependent on the frequency of outcome trending. EHR disease registries are used to capture, manage and provide access to condition-specific information for a specified group of patients to support organized clinical care [14,15]. Registries enable point of care integration with clinical practice guidelines, outreach to patients between visits to address care gaps and periodic evaluation of aggregate data and performance to manage a population [16]. Several registries have been developed at UCSDH and VASDHS for patients with conditions such as CKD, T2D and thrombotic disorders.

## 3. Collaborative Care Models

### 3.1. Pharmacist with Physician Oversight Care Model

#### 3.1.1. VASDHS Diabetes Intense Medical Management “Tune Up” Clinic

In 2009, a needs assessment identified over 9600 Veteran patients had a diabetes diagnosis and A1C values > 9% (per internal VASDHS VISN 22 Registry Data). In May 2009, the Diabetes Intense Medical Management (DIMM) “Tune Up” Clinic was created as a collaborative pharmacist–endocrinologist clinic to manage complex T2D patients and help them achieve clinical goals (e.g., A1C, blood pressure, lipid, weight goals) within 6 months. The clinical pharmacist specialist employs a short-term model that couples personalized clinical care with patient-specific diabetes education during an average of three 60 min visits over 6 months. After achieving goals, patients are referred back to their primary care provider (PCP). This allows a collaborative effort between the PCP and the pharmacist, frees up time during PCP visits to address other patient disorders, and provides patients with lifelong skills to manage their T2D and related metabolic disorders. The intended outcomes were to help PCPs and the medical center to achieve performance measures, avoid health system costs to treat diabetes complications, and prevent or delay patients from experiencing costly long-term complications of diabetes. Prior to implementation, the DIMM “Tune Up” clinic was reviewed, approved, and supported by key decision makers at the medical center: the Chief of Pharmacy, the Chief of Endocrinology, and the Chief of Internal Medicine, representing the PCPs.

Clinical issues, comorbidities, complications, socioeconomic and behavioral issues are integrated and assessed using the MTM Spider Web©, a visual tool developed for systematically creating a patient-centered clinical care plan [17]. The comprehensive nature of the tool helps ensure completeness and accuracy, as well as aiding patients to participate in shared decision-making while improving awareness and knowledge to assist in self-advocacy in their care. Strong emphasis is placed on adherence and educating and empowering patients to take control of their own diabetes without hypoglycemic events. Patients receive an individualized action plan and a personal medication record at each visit.

DIMM clinic outcomes demonstrate a 2.4% reduction in A1C compared to 0.8% in a PCP control group within 6 months, and glycemic control was achieved without significantly impacting medication regimen complexity [18,19]. A pharmacoeconomic analysis demonstrated that DIMM Clinic care resulted in $5287 savings per patient compared to usual PCP care, translating to a $9 to $1 return on investment [20]. Cost savings to the institution and reimbursement from insurance, as well as the strong metabolic outcomes, have justified continued clinic funding.

#### 3.1.2. UCSDH Diabetes Self-Management

The Diabetes Self-Management Program is an ambulatory care clinic providing diabetes self-management education for up to 350 patients annually with pre-diabetes or T2D. This program was developed, implemented and led by two pharmacists and a physician champion. Subsequently, it was accredited by the Diabetes Education Accreditation Program enabling insurance billing. The pharmacists are funded by the medical group and provide care following a CPA. Patients are referred by their UCSDH provider for group classes or individual visits at four different UCSDH outpatient clinics. The group education classes consist of two core, two-hour classes, each 1–2 weeks apart, covering seven diabetes self-management topics. For patients with cognitive deficits, hearing or visual impairment, a language barrier, or those requiring insulin initiation, individual 60 min visits are preferred. Services provided include medication reconciliation and the assessment of adherence, nutrition habits, and glucose meter results, with education tailored to patient’s specific needs. All patients set specific behavioral goals for themselves. Pharmacist recommendations are electronically sent to the referring provider after the visit. Telephone follow-up occurs 1–2 weeks following the classes or visits.

Annually, the program has over 500 office visits and 300 telephone follow ups. For group education, 45.8% patients attended one or two group meetings, and 13.2% attended three or four group meetings; the remaining patients were seen individually [21]. The mean decrease in A1C at 6 months was −0.6% compared to usual care −0.2%, *p* < 0.001, with no significant change in body mass index [21]. The program has also demonstrated a reduction in the number of patients with A1C > 8%, resulting in 55% achieving A1C <8% after 3 months and improved screening for A1C, with 57% of patients receiving two or more screenings per year. The outcomes have enabled continued funding of the program and the addition of a dietician (0.1 FTE) and scheduler (0.2 FTE).

#### 3.1.3. VASDHS Heart Failure Medication Management Clinic

Established in 2012, the HF medication management clinic objective was to reduce HF readmissions and improve outcomes in collaboration with the VA Advanced HF Team comprising cardiologists, nurse practitioners and nurses. The pharmacists’ scope of practice includes prescribing HF-related medications and ordering relevant laboratory and diagnostic studies. The advanced HF team establishes a diagnosis and initiates treatment, then referring patients for 30 to 60 min visits focused on medication management and follow-up. The pharmacist evaluates volume overload and worsening signs of congestion, titrates neurohormonal blockade to target doses, adjusts diuretics, assesses medication adherence and adverse effects. Typical medication titration visits can occur every 2–4 weeks, whereas patients requiring frequent diuretic adjustment may be seen weekly. The pharmacist works with the VASDHS quality improvement team to improve HF care and reduce hospital readmissions.

#### 3.1.4. SSPPS Partners in Medication Therapy (PMT)

PMT was established to provide medication therapy management services to self-insured employers, medical groups, and health plans. Services include optimizing medications and patient education on medication therapy for chronic diseases such as T2D, asthma, and cardiovascular disease. Partnerships with large third-party health plans generated opportunities for SSPPS APPs to work with different PCPs in Independent Practice Associations to provide telehealth visits. Patients were identified and referred to the APP based on gaps in therapy, adherence problems, or lack of treatment goal attainment detected by the health plan pharmacy claims data or the EHR. Clinical protocols were developed for chronic conditions such as HTN and T2D as well as therapeutic interchange to reduce drug costs. Under a CPA, APPs were authorized to initiate, modify, substitute, or discontinue agreed-upon medications according to the protocol and to authorize refills.

Using individual medical group EHR remote access, four APPs performed one-hour phone sessions to provide comprehensive medication management, order labs, document recommendations and communicate with PCP as needed. Each patient received an updated medication list and action plan. Follow up frequency was determined by patient-specific needs. Patients were discharged after goal attainment or failure to have detectable improvement after 6 to 12 months. In an initial four-month pilot program with St. Joseph Heritage Healthcare (Orange County, CA, USA), 756 patients were referred and screened with 61 resulting appointments.

### 3.2. Interprofessional (IP) Care Model

#### 3.2.1. UCSDH Chronic Kidney Disease (CKD)

In 2007, the CKD program was developed as an ambulatory clinic providing evidence-based IP care to delay CKD progression for approximately 400 patients with CKD Stages 2–5. UCSDHS funded the program as a separate cost center allowing financial outcomes including office visit volumes, medication revenue and expenses to be measured. The team consists of a nephrologist, pharmacist, nurse, dietitian and social worker. Patients see all team members at each visit, with new visits lasting approximately 90 min and return visits 60 min.

Under a CPA, the pharmacist conducts medication reconciliation, adherence assessment, and comprehensive medication management, facilitates access to medications, and provides patient education on changes to the medication therapy plan as well as the patient’s progress towards their health-related outcomes. The pharmacist optimizes medication therapy for hypertension, diabetes, proteinuria, hyperlipidemia, anemia, bone disease, hyperkalemia, metabolic acidosis and vaccinations. The pharmacist estimates kidney function and adjusts the doses of medications. The pharmacist orders prescriptions to the community pharmacy, medications to be administered in clinic, vaccinations and medications to be administered in the infusion centers. Patients receive one-on-one education during their clinic visit, are provided links to online education videos (developed by the CKD Program), and can attend a group class on dialysis modalities and kidney transplantation.

Implementation barriers included the expense of an IP team and low insurance reimbursement rates. Additionally, the pharmacist, social worker, and dietitian could not bill the insurers for their individual services, and the physician could not bill more complex office visits for services provided by the team. To minimize facility overhead expenses, the CKD program operates clinics in the same physical space as general nephrology clinics. Clinic volumes are at capacity, and the expansion of the program has been limited due to cost and available space. Despite challenges with expansion, this program has been sustained with a steady stream of new referrals.

This program was the first in the US to receive disease-specific certification in CKD from the Joint Commission, demonstrating high-quality care to patients [22]. Clinical and process outcomes routinely measured include but are not limited to blood pressure control, non-steroidal anti-inflammatory drug (NSAID) usage and counseling on avoidance, sick day management with angiotensin converting enzyme inhibitors or angiotensin receptor blockers, and referral for vascular access and transplantation as well as vaccination rates. With over 1000 office visits per year, the team achieved systolic blood pressure control in 58% of patients, with 0% of patients prescribed NSAIDs, 98% counselled to avoid NSAID products, and 64% viewing online CKD educational videos [23].

#### 3.2.2. UCSDH Oncology

Moores Cancer Center, the only National Cancer Institute-designated Comprehensive Cancer Center in San Diego, provides over 56,000 cancer patient visits annually including approximately 200 autologous and allogeneic hematopoietic stem cell transplants.

In 2014, the clinical pharmacy program was developed and implemented by a pharmacist in collaboration with a physician champion. Funding was provided by the cancer center for clinical services for two full days per week. The pharmacist initially developed a clinical practice in the thoracic medical oncology and the blood and marrow transplant departments. After one year of practicing, the pharmacist collaborated with leadership to expand the clinical pharmacy program by one full-time equivalent clinical pharmacy specialist in the head and neck/thoracic medical oncology ambulatory clinics.

The IP teams consist of oncologists, pharmacists, nurse case managers, mid-level providers (nurse practitioners and physician assistants), and medical assistants with the support of a social worker and a nutritionist on an as-needed basis. Clinical services provided by the pharmacist include medication management under a CPA, conducting medication reconciliation, oral chemotherapy adherence assessment, symptom management, immunization screening and ordering, therapeutic drug monitoring, oncology stewardship, and chemotherapy order preparation. The pharmacist provides literature support for the off-label utilization of cancer treatments. In addition, patients receive one-on-one education with the pharmacist or attend a group class focused on Chemotherapy and Immunotherapy Treatment that was developed and implemented by the pharmacist.

#### 3.2.3. UCSDH Pain and Palliative Care

The ambulatory and inpatient palliative care IP consult team is comprised of physicians, pharmacists, social workers, nurse practitioners and a chaplain. A description of the CPA development has been previously published [24]. In accordance with California code 4052, UCSDH palliative care pharmacists have independent prescriptive authority, with Drug Enforcement Agency (DEA) licensure and National Provider Index (NPI) status. Reasons for pharmacist consultation included physical symptom management, including but not limited to cancer-related pain, constipation, nausea and vomiting, fatigue, and insomnia.

Initial studies evaluating palliative care pharmacist contributions in the ambulatory care and inpatient settings have been positive [25,26,27]. Patients seen in clinic by a palliative care pharmacist versus patients who received standard of care showed trends for better pain control and health-related quality of life and function [26]. Medication prescribing changes by palliative care pharmacists commonly include a change in dose and/or the initiation of a new medication [27]. Positive trends were observed in pain control for patients with cancer over subsequent clinic visits [27]. Analgesic efficacy was improved by 17% and non-adherence was reduced by 13% [27].

Additionally, in the inpatient setting, the palliative care pharmacist provides opioid stewardship and guideline development for safe and effective analgesia. Prescribing trends are evaluated for safety and efficacy. Areas of focus include but are not limited to the prescription of fentanyl patches, patient-controlled analgesia, gabapentinoids, ketamine oral and intravenous infusion, lidocaine intravenous infusion, mexiletine, methadone, and naloxone [23,28,29,30,31,32,33,34,35,36]. Guidelines for the appropriate use of end-of-life opioids were developed [37]. Early access to the inpatient palliative care pharmacist resulted in a shortened length of stay, shortened length from admission to palliative care consult, and a positive impact on time from consult to discharge or death [25].

#### 3.2.4. UCSDH Solid Organ Transplantation

The Solid Organ Transplant program is a comprehensive IP center that provides evidenced-based, full-spectrum care from pre-transplant evaluation to post-transplant management for patients needing a kidney, pancreas, liver, lung, or heart transplant. The teams consist of transplant physician specialists (i.e., nephrologist, hepatologist, cardiologist, etc.), transplant surgeons, pharmacists, nurses, dietitians, and social workers. In 2004, the bylaws of the United Network for Organ Sharing were amended to include pharmacists as necessary providers on the transplant team [38]. There are eight dedicated transplant pharmacists, including two faculty pharmacists who each devote 15% effort to ambulatory patient care and receive salary support from the UCSDHS Center for Transplantation. Revenue generated by improving patient utilization of discharge and ambulatory UCSDH pharmacies has led to expansion and continued salary support for transplant pharmacists.

Similar to other services in the health system, transplant pharmacists initiate, discontinue, change, and monitor drug therapy under a CPA. Working closely with transplant physicians and surgeons, pharmacists manage a patient’s immunosuppressive therapies, as well as treatments for complications of immunosuppression, other associated medical conditions, and preventative therapies. The pharmacists conduct medication reconciliation, adherence assessment, a focused assessment of immunosuppression, MTM for various complications of transplantation, order laboratory tests, immunizations, facilitate access to medications and provide comprehensive patient education. In 2018, approximately 95 kidney, 41 liver, 56 heart, and 23 lung transplants were performed, and long-term follow-up was provided.

#### 3.2.5. UCSDH Transitions of Care (TOC)

The TOC program aims to optimize care and reduce hospital readmissions by providing hospital and ambulatory care continuity services for approximately 7800 high-risk patients within UCSDH. Funding support for the TOC program was initially driven by Medicare incentive programs such as Delivery System Reform Incentive Payment (DSRIP) and the Community-Based Care Transitions Program (CCTP) to improve care transitions for high-risk patients. The TOC pilot program was implemented by a pharmacist to target patients with advanced stages of HF. Following the conclusion of the DSRIP and CCTP projects, funding from UCSDH was justified with documented interventions from the pilot programs. The TOC pharmacist service has since expanded to include other populations (e.g., HIV) and select groups of managed care patients. An SSPPS faculty member joined the TOC program with UCSDH funding support of 40% in an effort to assist with the increased breadth of patient services.

TOC pharmacists work under a CPA with prescribing authority to initiate, adjust, monitor, or discontinue medication therapy for HF and related conditions in hospital and ambulatory clinics. Inpatient TOC pharmacists make rounds daily with an IP team, conduct admission and discharge medication reconciliation, assess adherence, collaborate with the medical team on medication therapy, facilitate access to medications, and provide comprehensive patient education. Ambulatory TOC pharmacists perform phone-call follow-up and focus on post-discharge patients in-clinic to conduct medication reconciliation, assess adherence, perform MTM for HF and related conditions, facilitate medication access, and provide patient education.

Given the expense of pharmacist salaries and the inability to bill insurers for pharmacist services, funding remains the primary barrier to expanding the TOC program. Funding for pharmacy technicians and longitudinal TOC introductory pharmacy practice experiences for pharmacy students have been implemented to maximize budget resources. Approximately 70% of patients require a pharmacist intervention, with an average of two interventions per patient.

### 3.3. Pharmacist-Physician Team Care Model

#### 3.3.1. UCSDH Family Medicine Clinics

The UCSDH Family Medicine Clinics provide services at three locations throughout San Diego County to patients from pre-conception to end-of-life care. Since 2010, two clinical pharmacists have provided chronic disease management under a CPA. Patients are identified by the PCP and are referred for a “shared” collaborative visit with a pharmacist and a family medicine physician. During each visit, the pharmacist conducts medication reconciliation and reviews the patient’s past medical history, relevant family and social histories, risk factors, allergies, medications, and lab results. Pharmacists manage anticoagulation, diabetes, HTN, hyperlipidemia, tobacco cessation, and immunizations. A care plan is developed with the patient that includes treatment goals and education. The plan is communicated with the physician, who also meets with the patient to address any further questions or health problems. Based on the complexity of the visit, insurance is billed with the physician signing off on the care plan. The PCP is also notified of the care plan. Patients are seen in 30 min appointments with regular follow-up, accounting for over 1000 annual office visits.

Ongoing and regular physician referrals remain a challenge. Patients may also be identified through EHR registries or dashboards and then scheduled for a pharmacist visit, although patients are more likely to attend visits when suggested by their PCP. Additionally, since the physician paired with each pharmacist is different at each session, it is not always guaranteed that a patients’ own PCP will be the physician during their shared visit. The physician sees his/her own limited set of patients for the session, and so challenges arise with respect to timing and visit duration, impacting the patient and provider experience. While these types of visits do increase IP collaboration and allow for more complex visits and reimbursement, there have been discussions about moving toward a more independent-pharmacist model within the clinics.

Clinical outcome measures being implemented include improved A1C, anticoagulation time in the therapeutic range, the achievement of goal blood pressure, and the primary and secondary prevention of coronary artery disease and stroke. This particular program was selected as one of three in 2018 by the Centers for Disease Control and Prevention for an evaluability assessment site visit as a program routinely using innovative strategies in hypertension care [39].

#### 3.3.2. Geriatric Community Partnerships

In 2015, UCSDH was awarded a grant from the federal Health Resources Services Administration Geriatrics Workforce Enhancement Program (GWEP) of the Department of Health and Human Services to train and research novel approaches to providing older adult care. One particular focus of this proposal was the incorporation of a clinical pharmacist in a primary care clinic to provide direct-patient care for underserved populations via a partnership with the Medicare–Medicaid-funded Program of All-Inclusive Care for the Elderly (PACE) Clinic in San Ysidro, CA, and to train and embed a pharmacist fellow in the clinic to perform comprehensive medication management under a CPA [40]. The funding also supported a part-time student pharmacist to assist with clinic provider education on potentially inappropriate medications, polypharmacy, medication regimen complexity, program development, and participation in required rapid-cycle quality improvement projects [41]. The patient population was 90% percent Spanish-speaking, with limited health literacy and, as Medicaid qualifiers, they were low-income patients with an average of 14 medications per patient. The pharmacist conducts transitions of care medication reconciliation, manages anticoagulant dosing, initiates immunizations, reduces the use of inappropriate medication and high-cost medications, and provides medication usage recommendations to center management [42]. The clinic has continued its funding of a full-time clinical pharmacist.

#### 3.3.3. UCSDH Psychiatry

UCSDH Outpatient Psychiatry Services include general psychiatry and specialty clinics in mental health such as college mental health, child and teen psychiatry, addiction treatment, treatment-resistant depression, women’s reproductive mental health, sexual health, early psychosis, and senior behavioral health. The clinics employ numerous health professionals to provide a full range of outpatient psychiatric services, including intakes, individual and group psychotherapy, pharmacotherapy, and psychoeducation. Established in 2008 [43], the pharmacist clinic has experienced between approximately 150–300 annual office visits [43]. The patient population varies from county-funded patients with severe mental illness to public and private funding for mood disorders, anxiety disorders, PTSD, and ADHD. The Department of Psychiatry provides funding for 10% work for the psychiatric pharmacist’s clinic time.

During each visit, the psychiatric pharmacist spends approximately 30–45 min providing psychotropic medication management under a CPA. The pharmacist conducts medication reconciliation, assesses medication adherence and adverse effects, evaluates medication dosing, assists with refills and insurance authorizations, and makes referrals to other services. The pharmacist and attending psychiatrist develop the therapeutic plan and then discuss the plan with the patient. The pharmacist has a DEA license, which allows the initiation and modification of controlled and non-controlled medications. Recently, the pharmacist coordinated the initiation of a new, long-acting, injectable antipsychotic program to improve patient access and continuity of care.

Implementation challenges include funding for the pharmacist’s work, insurance billing, shared visit scheduling, and regulatory requirements for documentation. Program outcomes include patient encounter volumes, top patient diagnosis, and pharmacist interventions. Future program outcomes will include symptom improvement using standard rating scales and required medication monitoring documentation.

## 4. Discussion

In this paper, we describe collaborative pharmacist–physician practice models that provide clinical care to patients with complex disease states. The pharmacists not only focus on the optimization of care for their respective specialties but also provide comprehensive care for comorbid conditions and complications. Notably, the pharmacists provide a large scope of services which encompass medication therapy management, including but not limited to medication reconciliation, initiating and adjusting medications, the ordering and monitoring of laboratory tests, performing physical examination, assessing and optimizing adherence, promoting healthy lifestyles, and providing education. These services were delivered using different care models integrating the pharmacist within IP teams or partnering with physicians.

The type of practice model chosen was based on the disease state, patient complexity, needs assessment, strategic goals, and financial viability. We summarized the advantages and disadvantages for the described care models (Table 1). For example, the DIMM clinic model is a pharmacist with physician oversight model since evidence-based medication optimization to improve glycemic control could be most efficiently delivered by the pharmacist provider. However, in psychiatry, the pharmacist–physician team care model was chosen due to patient complexity and the need for an individualized approach to management that considers numerous patient factors.

Importantly, the majority of these care models have been funded by the affiliated health systems, and a few are funded by grant mechanisms. This was made possible by creating goals that align with the health system priority initiatives and recruiting faculty whose expertise could help to achieve these goals. A business case was developed by reviewing published literature, identifying potential quality outcomes and direct and indirect financial metrics. Several studies have been published demonstrating the financial benefit of pharmacist care services in a variety of care environments from community pharmacy to ambulatory care clinics and to hospital acute care environments. In a study examining the impact of the community pharmacist-initiated management of uncomplicated urinary tract infections, pharmacist care was associated with lower costs ($72.47) when compared to family ($141.53) and emergency ($368.16) physician-initiated care, estimated to result in significant long-term net total savings of $51 million [44]. Ourth and colleagues conducted a cost-effectiveness study of clinical pharmacists managing diabetes and found a negative cost per quality of life adjusted years after 3 years, suggesting that it is cost-effective to incorporate pharmacists into diabetes care teams [45]. Similarly, studies of transitions of care programs have demonstrated significant cost savings. Ni and colleagues documented average cost savings of $2139 in the first 180 days after discharge [46].

The practices described in this paper proved to be sustainable with continued funding for long periods of time despite cessation of grant funding. Moore and colleagues found that after initially funding pharmacist services through grant mechanisms and demonstrating quality care, the services could be continued and expanded through funding by accountable care organizations [47]. We demonstrate that clinical faculty can effectively provide comprehensive pharmacist services to a large breadth of complex patient populations across multiple practice settings in affiliated health systems. Our programs position pharmacists to obtain APP status in the future given their collaborative practice meets necessary criteria for APh licensure.

We stated that the outcome measurement of these pharmacist care models varies with some practices in the design phase and others measuring patient volume to robust clinical and process outcome dashboards. Successful programs measure meaningful outcomes that demonstrate the return on investment for the health system. Many of our programs have developed significant improvements in clinical and process outcomes. We demonstrated that pharmacist-delivered diabetes care resulted in A1c reductions of 2.4%, compared to 0.8% for standard of care, with significant cost savings. The pharmacist within the IP CKD care team focused on reducing the number of patients prescribed an NSAID to 0% and counseling over 98% patients on NSAID avoidance. In palliative care, pharmacist interventions resulted in 17% improved medication efficacy and a reduction in non-adherence by 13%. Transitions of care pharmacists demonstrated a significant reduction in hospital readmissions with cost savings to the institution. These clinical and process outcomes have been revised as needed for continuous quality improvement, with the retirement of outcomes at goal and new outcomes identified. In some instances, new outcomes or strategies were developed for financial sustainability. These results have been disseminated at national and international IP conferences, or published in journals with broad readership. This dissemination is critical to allow for these care models to be easily replicated at other institutions.

The value of pharmacist services we described has received notable recognition from government agencies, accreditation bodies, and professional societies. From these various models of pharmacist services, we identified some common facilitators. These included a gap in care that could be met with pharmacist services, a linkage between clinical and financial outcomes, a physician champion to support proposals, and the strategic recruitment of faculty with expertise in these growth areas at affiliated health systems.

Program design and implementation always comes with challenges; space and funding are well-recognized resource barriers. However, our collective approach has been to share our faculty experiences and to brainstorm funding mechanisms and develop successful space propositions such as colocation. The colocation of interprofessional team members in a shared work space and elimination of private physician work offices has been shown to improve team communication, cohesiveness, and team primacy [48]. An important facilitator of funding challenges is the joint appointment of the UCSDH Chief Pharmacy Officer as a clinical faculty member that provides a critical advantage for the development of new clinical services at UCSDH. Our future directions include the development of new clinical faculty practice settings in geriatrics and infectious disease within the affiliated health systems.

## 5. Conclusions

In conclusion, the expansion of pharmacist services delivered by clinical faculty has several benefits to affiliated health systems: (1) significant improvements in patient care quality, (2) access to experts in specialty areas, and (3) the dissemination of outcomes and national and international recognition, increasing the visibility of the health system.

## Figures and Tables

**Table 1:** Advantages and disadvantages of different pharmacist care models.

Care Model	Advantages	Disadvantages
Pharmacist with physician oversight	Autonomy in clinical decision making. Efficient model for optimizing medication management using evidence-based guidelines. Improves public recognition of advanced pharmacist provider roles.	Can only be applied to patients with established diagnosis who mainly require optimization of medications. Complex patients requiring additional diagnostic testing may not be appropriate. Pharmacist may feel a disconnect from physician collaborator. Dependent on referrals from physician collaborators. Low reimbursement on visits in the United States.
Interprofessional	Best suited for complex disease states or patients with numerous comorbidities. Patient convenience-comprehensive care delivered in one single office visit. Greater ability to achieve target clinical outcomes with multi-prong management (e.g., lifestyle modification, medications, social interventions) Higher reimbursement since physician provider bills for visits.	Requires greater coordination of team members during visits to ensure efficiency. Less time for each health professional to spend with patient. Longer visit duration for patients. Longer visits may result in lower patient volumes. Interprofessional team is resource intensive.
Pharmacist-Physician Team	Can manage established and newly diagnosed patients who may require additional diagnostic testing. Enhances care provided in physician only visits due to optimization of drug therapy. Easier to coordinate team members. Higher reimbursement since physician provider bills for visits.	Less pharmacist autonomy Longer visits Dependent on referrals for patient volume.
